# Astrocytes infected with *Chlamydia pneumoniae* demonstrate altered expression and activity of secretases involved in the generation of β-amyloid found in Alzheimer disease

**DOI:** 10.1186/s12868-019-0489-5

**Published:** 2019-02-20

**Authors:** Zein Al-Atrache, Danielle B. Lopez, Susan T. Hingley, Denah M. Appelt

**Affiliations:** Department of Bio-Medical Sciences, Center for Chronic Disorders of Aging, Philadelphia College of Osteopathic Medicine, 4170 City Avenue, Philadelphia, PA 19131 USA

**Keywords:** Alzheimer disease, Neuroinflammation, Neurodegeneration, Amyloid, Astrocytes, Secretase, BACE1, Chlamydia pneumoniae, Pathogens

## Abstract

**Background:**

Epidemiologic studies strongly suggest that the pathophysiology of late-onset Alzheimer disease (AD) versus early-onset AD has environmental rather than genetic causes, thus revealing potentially novel therapeutic targets to limit disease progression. Several studies supporting the “pathogen hypothesis” of AD demonstrate a strong association between pathogens and the production of β-amyloid, the pathologic hallmark of AD. Although the mechanism of pathogen-induced neurodegeneration of AD remains unclear, astrocytes, a key player of the CNS innate immune response and producer/metabolizer of β-amyloid, have been implicated. We hypothesized that *Chlamydia pneumoniae* infection of human astrocytes alters the expression of the amyloid precursor protein (APP)-processing secretases, ADAM10, BACE1, and PSEN1, to promote β-amyloid formation. Utilizing immunofluorescent microscopy, molecular, and biochemical approaches, these studies explore the role of an intracellular respiratory pathogen, *Chlamydia pneumoniae*, as an environmental trigger for AD pathology. Human astrocytoma cells in vitro were infected with *Chlamydia pneumoniae* over the course of 6–72 h. The gene and protein expression, as well as the enzymatic activity of non-amyloidogenic (ADAM10), and pro-amyloidogenic (BACE1 and PSEN1) secretases were qualitatively and quantitatively assessed. In addition, the formation of toxic amyloid products as an outcome of pro-amyloidogenic APP processing was evaluated through various modalities.

**Results:**

*Chlamydia pneumoniae* infection of human astrocytoma cells promoted the transcriptional upregulation of numerous genes implicated in host neuroinflammation, lipid homeostasis, microtubule function, and APP processing. Relative to that of uninfected astrocytes, BACE1 and PSEN1 protein levels were enhanced by nearly twofold at 48–72 h post-*Chlamydia pneumoniae* infection. The processing of APP in *Chlamydia pneumoniae-*infected astrocytes favors the pro-amyloidogenic pathway, as demonstrated by an increase in enzymatic activity of BACE1, while that of ADAM10 was decreased. Fluorescence intensity of β-amyloid and ELISA-quantified levels of soluble-APP by products revealed temporally similar increases, confirming a BACE1/PSEN1-mediated processing of APP.

**Conclusions:**

Our findings suggest that *Chlamydia pneumoniae* infection of human astrocytes promotes the pro-amyloidogenic pathway of APP processing through the upregulation of expression and activity of β-secretase, upregulated expression of γ-secretase, and decreased activity of α-secretase. These effects of astrocyte infection provide evidence for a direct link between *Chlamydia pneumoniae* and AD pathology.

**Electronic supplementary material:**

The online version of this article (10.1186/s12868-019-0489-5) contains supplementary material, which is available to authorized users.

## Background

Alzheimer Disease (AD) is a chronic, progressive neurodegenerative illness regarded as the most common cause of dementia, which afflicts 46.8 million people worldwide—a number predicted to quadruple by the year 2050 [1]. AD also ranks among the top 10 causes of death in the US that can be neither prevented nor cured. Several investigations have aimed at deciphering etiologies that suggest the multiple causes or triggers of AD pathology [2, 3]. AD is diagnosed as one of two classifications: early-onset or familial AD, abbreviated EOAD or FAD, or late-onset or sporadic AD, abbreviated LOAD or SAD. Familial AD manifests symptoms at < 65 years of age. These cases are predominantly genetic and account for only 5% of all cases of AD. The majority of AD patients that present with symptoms at > 65 years of age are grouped into the category of late-onset AD. Numerous genome-wide association studies have identified that certain risk-associated alleles are expressed differently in patients suffering from EOAD/FAD versus LOAD/SAD [4, 5]. However, the etiology of SAD pathology, unlike that of FAD, is considered multifactorial rather than strictly genetic.

In 1992, Hardy and Higgins [6] first introduced the amyloid cascade hypothesis to explain the etiology of AD, which proposes that beta-amyloid (Aβ) results from the enzymatic processing of amyloid precursor protein (APP). The amyloid cascade hypothesis claims that the neurotoxicity triggered by Aβ initiates related pathologic processes such as formation of extracellular senile plaques, which is one of the characteristic hallmarks of AD. Senile plaques are composed of oligomerized Aβ and are the pathologic findings in FAD and SAD brains. In its monomeric form, Aβ is a 39–42 amino acid peptide fragment derived from the sequential cleavage of APP, a large, type I transmembrane protein. APP processing can occur in two pathways, the non-amyloidogenic pathway or the pro-amyloidogenic pathway. The initial APP-processing event is catalyzed predominantly by the α-secretase, a disintegrin and metalloproteinase-10 (ADAM10) in the non-amyloidogenic pathway [7], and the β-secretase, β-site APP cleaving enzyme 1 (BACE1), in the pro-amyloidogenic pathway [8, 9]. A second cleavage event is performed by a γ-secretase, a complex containing 4 subunits: presenilin 1 or 2 (PSEN1 or PSEN2), as the catalytic subunit, nicastrin (NCSTN), anterior pharynx defensive phenotype 1 (APH1) and presenilin enhancer-2 (PEN2) [10, 11]. Cleavage of APP by ADAM10 or BACE1 releases the soluble amino terminal products, soluble APP-α (sAPPα) or soluble APP-β (sAPPβ), respectively, and carboxy terminal fragments, C83 and C99, respectively. In the non-amyloidogenic pathway, C83 is further cleaved by PSEN into the APP intracellular domain (AICD) and a p3 peptide, a non-toxic form of amyloid. Whereas in the amyloidogenic pathway, PSEN cleaves C99 into AICD and Aβ fragments, of which the 42 amino acid fragment (Aβ_1-42_) is considered the most neurotoxic [12]. Due to its hydrophobic characteristics, Aβ_1-42_ acts as a nidus for seeding additional Aβ peptide fragments, thereby facilitating large, extracellular aggregations of Aβ [13, 14].

In the context of SAD pathogenesis, the well-established mutations of APP and PSEN that promote enhanced pro-amyloidogenic processing of APP in FAD are not implicated; rather, exogenous stimuli, such as environmental toxins or infectious pathogens that may alter their overall expression are implicated [15]. These exogenous stimuli trigger the activation of both neuronal and non-neuronal cells with subsequent release of pro-inflammatory cytokines and activation of intracellular signaling pathways [16, 17]. As a result of these types of stressors, activated glial and neuronal cell models have demonstrated increased transcriptional expression, and/or altered activity of ADAM10, BACE1, and PSEN1 [18–20]. The etiology of neurodegeneration in SAD may therefore result from, at least in part, the effects of exogenous stimuli on the expression of APP-processing secretases.

One such stimulus that has garnered significant support as a potential trigger of SAD pathology is infection of the CNS by various pathogens. Several pathogens that have been implicated in SAD include cytomegalovirus, herpes simplex virus type 1, *Borrelia burgdorferi*, and *Chlamydia pneumoniae* (*Cpn*) [21, 22]. The role of *Cpn* in SAD pathology has been illustrated at both the epidemiologic and cellular levels. This relationship was first cited in the seminal study by Balin et al. [23] that demonstrated that metabolically active *Cpn* was found by immunohistochemical, electron microscopic, and PCR techniques to be localized to areas of AD pathology in 17 of 19 post-mortem AD brains compared to 1 of 19 non-AD control brains. Another study validated the presence of viable *Cpn* in 80% of AD brains (versus 11.1% of age-matched controls) via multiple methods including in situ hybridization and PCR analysis of *Cpn*-specific targets [24]. Additional evidence for a causal relationship between *Cpn* and AD was demonstrated through intranasally inoculating the non-genetically manipulated BALB/c mouse with *Cpn* isolates from AD brains [25]. In that study, Aβ deposits associated with *Cpn* infection were found in brain areas that are typically affected in AD such as the hippocampus, the dentate gyrus and the amygdala. These plaques were surrounded by reactive astrocytes and, at times, encircled brain vasculature, suggesting the presence of cerebral amyloid angiopathy.

Epidemiologic assessments of *Cpn* and other infectious burdens in control versus AD brains show a correlation between infection and AD [21, 22, 24]. This evidence supports the hypothesis that the chronic neuronal and glial cell dysfunction visualized in the brains of SAD patients may be derived from early-acquired CNS infection by *Cpn* and similar intracellular pathogens with the potential to persist over time and reactivate from latency or persistence.

An investigation into aberrant APP metabolism and Aβ accumulation in the setting of inflammation needs to include an analysis of the role of astrocytes, the most abundant glial cells in the CNS. A common observation among studies investigating *Cpn* in post-mortem AD brains [23] and brains of *Cpn*-inoculated BALB/c mice [25] was the colocalization of *Cpn* and GFAP-labeled astrocytes, suggesting astrogliosis in response to *Cpn* infection. It is interesting to note that glial activation in AD patients is not uncommon, as revealed by PET imaging during the pre-symptomatic stages of AD, and is shown to correlate with the initial signs of Aβ accumulation [26]. Animal models and in vitro studies indicate that astrocytes respond to immune- and AD-associated triggers, such as TNF-α, IFN-γ, IL-1β, bacterial lipopolysaccharide and Aβ by releasing cytokines and modifying the expression and activity of APP processing enzymes, which in turn exacerbate neuroinflammatory and neuropathological changes in the AD brain [19, 20, 27–30]. These findings support the contention that reactive astrocytes contribute to the neurodegeneration and loss of cognition observed in AD. Therefore, investigating the effect of infection by *Cpn* on the processing of APP by astrocytes is invaluable in modeling potential mechanisms by which *Cpn* may trigger sporadic AD pathology, especially over time.

This study is aimed at investigating the effects of infection by *Cpn* on genes and the gene products involved in the processing of APP to produce Aβ, which is a major characteristic of AD pathology. By examining the effect of *Cpn* infection on validated pathways of astrocytic APP processing, this study provides evidence to support that AD pathology is recapitulated by infection with *Cpn*. This investigation explores how the expression and activity of APP-processing machinery, as defined by the amyloid cascade hypothesis, is altered as a result of *Cpn* infection of STTG1 human astrocytoma cells. The STTG1 human astrocytoma cell line has been suggested to be a valuable in vitro model for AD and its experimental therapies. This is due to STTG1’s heterozygous expression of the ApoE ε3/4 gene, its active participation in the pro-inflammatory cascade, and ability to both synthesize and breakdown Aβ [31–34]. Therefore, this in vitro model of *Cpn* infection of the CNS not only enhances our understanding of pathologic AD mechanisms, but also brings to light new research avenues investigating “the pathogen hypothesis” for early diagnosis and treatment of sporadic AD.

## Methods

### Cell culture and infection with *Chlamydia pneumoniae*

The human astrocytoma cell line CCF-STTG1 (CRL-1718) was obtained from American Type Culture Collection (ATCC, Rockville, MD, USA). Cells were grown at 37 °C and 5% CO_2_ as a monolayer in culture medium RPMI-1640 (ATCC, 10-2001) supplemented with 10% (v/v) fetal bovine serum (FBS) in culture flasks (Corning Cell Culture Treated Flasks). Cells were trypsinized (Thermofisher) and transferred to 12 or 6 well polystyrene plates (Corning^®^ CellBIND^®^) in culture medium for the western blot and RT-PCR experiments. For the immunocytochemistry experiments, cells were grown on 18.5 mm glass coverslips (neuvitro, GG-18-1.5-pre) in sterile 12-well plates. For cell infection experiments, 50% of conditioned growth media was removed and *Cpn* strain AR39 (ATCC, 52592) at MOI = 1 was added to 5 × 10^4^ to 1 × 10^5^ cells/well. To minimize variability *Cpn* lot number was held constant throughout experiments and each time point for a given experiment was inoculated on the same day. After centrifugation at 300×*g* for 30 min at RT, fresh growth media was added and cells were incubated for 6, 24, 48, and 72 h. Uninfected cells used as a negative control were processed in parallel with *Cpn*-infected cells. This procedure describes the preparation for one biological replicate. Each timepoint was repeated to achieve samples in biological triplicate, for which each was run in at least technical triplicate for western blot analysis, ELISA, immunocytochemistry, and molecular studies. Additional information of sample sizes for each study is included in its respective figure legend.

### Immunocytochemistry

Cells grown on sterile 18.5 mm glass coverslips were incubated with the following primary antibodies: anti-Aβ_1-42_ at 1:500 (Synaptic Systems, 218703); anti-ADAM 10 at 1:100 (abcam ab39180), anti-BACE1 at 1:500 (abcam, ab10716), anti-presenilin-1 at 1:500 (ProSci 4203). Secondary antibody was used at 1:500 (Alexa Fluor^®^ 594); FITC-conjugated chlamydial antibodies (Fitzgerald, 61C75-A and 60C19) were used at 1:100 to visualize the infection. BD Perm/Wash™ was used to as the antibody diluent and cell wash buffer. Coverslips were mounted on glass slides using FLUORO-GEL II with DAPI (EMS, 17985-50). Images were acquired using an Olympus FV1000 laser scanning confocal microscope with a 60×, 1.4NA oil immersion objective lens and FluoView 1000 software. For cell counts, images were acquired at 40× using the Nikon Eclipse 90i epi-fluorescence microscope.

### Alzheimer disease RT-PCR array

Cells were harvested and RNA was isolated in biological triplicate from *Cpn* infected and uninfected astrocytes at each timepoint post-infection. Purified RNA was reverse-transcribed using RT^2^ First Strand Kit (Qiagen, 330401). To ensure that the comparisons of gene expression were valid for each timepoint post-infection, an equal amount of RNA template from uninfected and *Cpn*-infected cells within each timepoint was used for cDNA synthesis. cDNA was used to profile 84 different genes included in the Human Alzheimer Disease RT^2^ Profiler ™ PCR Array (Qiagen, PAHS-057ZC). Web-based PCR array data analysis software provided by Qiagen was used to collectively analyze raw C_t_ values for each AD-related gene included in the assay [35]. Human β-actin was automatically chosen by the analysis software as the housekeeping gene for standardization. Once each assay was normalized to β-actin, ΔC_t_ values for each gene of interest in *Cpn*-infected cells were compared to that of uninfected cells to obtain a fold change between gene of interest expression in uninfected cells and that of *Cpn*-infected cells for each timepoint. Statistical significance in fold change values were determined by the Qiagen online analysis software, which uses a two-tailed student’s *t* test to compare gene expression in infected and uninfected samples. Statistically significant changes in expression of AD-related genes are listed in Additional File 1.

### Western blot analysis

At the indicated timepoints post-infection, uninfected and *Cpn*-infected cells were lysed using 1 × RIPA lysis buffer (EMD Millipore, 20-188) supplemented with 1× protease inhibitor (Halt ™ Protease Inhibitor 100×, Thermo Scientific, 78430). Cell lysates were homogenized mechanically with mortar and pestle and through ice-cold sonication before resolving on 4–20% precast polyacrylamide gels (Bio-Rad, Mini-PROTEAN^®^ TGX™ gels, 456-1094) using 1× Tris/glycine/SDS running buffer (Bio-Rad, 161-0732). Gels were transferred onto nitrocellulose membrane (iBlot^®^ transfer stack, Life Technologies, IB3010-02). Membranes were then washed with wash buffer (Pierce ^®^ Fast Western Blot Kit, Thermo Scientific, 35050) and labeled (24 h at 4 °C with gentle agitation) with primary antibodies diluted in antibody diluent (Pierce ^®^ Fast Western Blot Kit, Thermo Scientific, 35050). The following primary antibodies were used: anti-ADAM10 at 1:400 (Santa Cruz Biotechnology, sc-48400), anti-BACE1 at 1:500 (abcam, ab108394), anti-presenilin-1 at 1:500 (abcam, ab76083), anti-chlamydial antibody at 1:200 (Fitzgerald 10C27B), and to label the housekeeping protein of interest, anti-β-actin at 1:500 (Santa Cruz Biotechnology, sc-8432). The following secondary antibodies were used: goat anti-mouse and anti-rabbit conjugates at 1:500 (Bio-Rad, 170-5046 and 170-5047, respectively). SuperSignal^®^ West Pico Chemiluminescent Substrate (Thermo Scientific, 34080) was then applied to the membranes and visualized using Bio-Rad VersaDoc Imaging System 4000MP. Densitometry analysis was conducted using FIJI software [36]. ADAM10, BACE1, and PSEN1 expression were assessed using the same samples, ensuring that the expression of each of these proteins could be accurately compared to each other. The expression of each protein of interest was quantified in 5–7 total samples. To determine statistical significance between protein levels derived from *Cpn* infected and uninfected cells, student’s *t*-test was conducted on the optical density values of each protein of interest normalized to that of β-actin as a loading control.

### Quantitative analysis of Aβ_1-42_ using immunofluorescence

Following immunolabeling with anti-Aβ_1-42_ as previously described, thirty 2 μm Z-stack images were acquired across 3 separate coverslips (approximately 10 cells per coverslip) to ensure that representative populations of cells were captured and that the entire 3-dimensional depth of labeled Aβ was included in the analysis. Using FIJI software [36], each Z-stack image was separated into its three individual channels; DAPI and FITC were removed at this point and analysis was performed on the TRITC channel. From each analyzed image, a single 2D composite image was resolved to represent the maximum fluorescence intensity of each pixel of each 0.2 μm slice. Threshold fluorescence level was defined to include all Aβ labeling within the area of the cell. Mean fluorescence intensity of Aβ fluorescence exceeding the threshold fluorescence level was obtained for each imaged cell and a student’s *t*-test was conducted to determine statistical significance of Aβ_1-42_ fluorescence intensity between infected and uninfected cells for 24, 48 and 72 h timepoints.

### Quantitative analysis of soluble APPα and APPβ using Meso Scale Discovery (MSD) ELISA

At the indicated timepoints post-infection, conditioned media from *Cpn*-infected and uninfected cells was removed and stored at − 80 °C. One milliliter of the conditioned media was thawed and concentrated using Eppendorf Vacufuge Plus at 45 °C. The MSD 96-well MULTI-SPOT sAPPα/sAPPβ assay was performed as directed by the manufacturer. Inter- and intra-assay %CV are listed in Additional File 2. Concentration readings of each individual sample (3 for each timepoint post-infection and infection status) of sAPPβ were divided by total sAPP (sAPPα + sAPPβ) to obtain a ratio of sAPPβ to total sAPP in the conditioned media of uninfected and *Cpn*-infected treatment groups.

### ADAM10 and BACE1 Activity Assay

AnaSpec Sensolyte 520 ADAM10 and BACE1 Activity Assay Kits (AS72226 and AS71144, respectively) were used as a fluorimetric method for determining ADAM10 and BACE1 activity in *Cpn*-infected and uninfected astrocytes. After 48 hpi, cells were counted, (data not shown in results) harvested and placed into pre-chilled microcentrifuge tubes with pre-packed pestle for homogenization. Cells were washed with ice-cold PBS, and pelleted at 4 °C and 800×*g* for 10 min (4 samples for each treatment group). Mechanical and liquid nitrogen snap-freeze homogenization of fresh lysate allowed for retrieval of lysate without compromising enzymatic activity. Each enzyme assay was performed as directed by the manufacturer. Each sample was assayed in technical duplicate. Completed assays were loaded in a black, clear bottom plate and incubated at 37 °C for 1 h, then analyzed at Ex/Em 490/520 using a Fluoroskan Ascent FL microplate fluorometer. Background fluorescence of assay buffer was subtracted from final fluorescence measurements and each final measurement was normalized based on the protein concentration as determined by BCA protein assay (Pierce, 23225).

## Results

### *Chlamydia pneumoniae* infects STTG1 human astrocytoma cells in vitro and is maintained through 72 h post infection

**Fig. 1:**
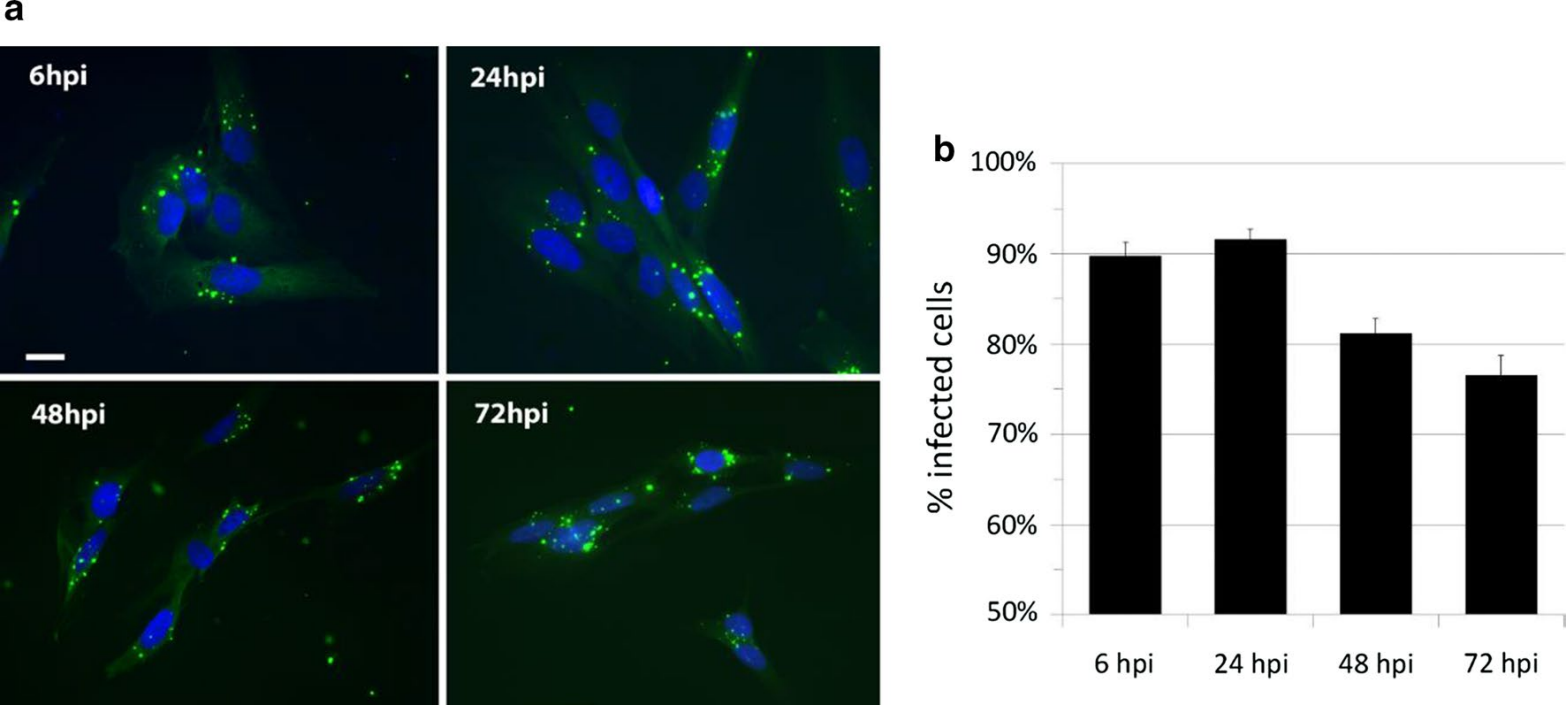
*Chlamydia pneumoniae* infects human astrocytes in vitro. STTG1 human astrocytes infected with Cpn strain AR39 at an MOI of 1 demonstrated a diffuse punctate labeling of Cpn (green) from 6 to 72 hpi. Nuclei are labeled with DAPI (blue). Scale bar represents 20 μm (**a**). Infected versus uninfected cell counts were averaged across approximately *N* = 2000–2500 cells per timepoint and in biological triplicate across two independent infections. Numerical data is expressed as percent infected cells (**b**). Percentages of infected cells were significantly different between 6 hpi versus 48 hpi and 72 hpi, 24 hpi versus 48 hpi and 72 hpi. Comparisons between populations were determined through one-way ANOVA, where significance was defined as *p* < 0.05, and confirmed using Tukey HSD post hoc analysis. Error bars represent standard deviation of the mean

As visualized via confocal microscopy, the respiratory strain of *Cpn*, strain AR39, robustly infects STTG1 astrocytoma cells in vitro and persists 72 h post-infection (hpi) (Fig. 1a). Percent infected cells, averaged across approximately 2000–2500 cells per timepoint, is shown in Fig. 1b. Percentages of infected cells were significantly different between 6 hpi versus 48 hpi and 72 hpi, 24 hpi versus 48 hpi and 72 hpi. This was determined through conducting a one-way ANOVA, demonstrating *p* < 0.05, and confirmed with Tukey HSD post hoc analysis. These data indicate that *Cpn* infects human astrocytoma cells within 6 hpi and appears to remain viable within these cells for at least 72 h, although the numbers of infected cells decrease after 24 hpi.

### *Chlamydia pneumoniae* infection of human astrocytes alters the transcript expression of AD-related genes

**Fig. 2:**
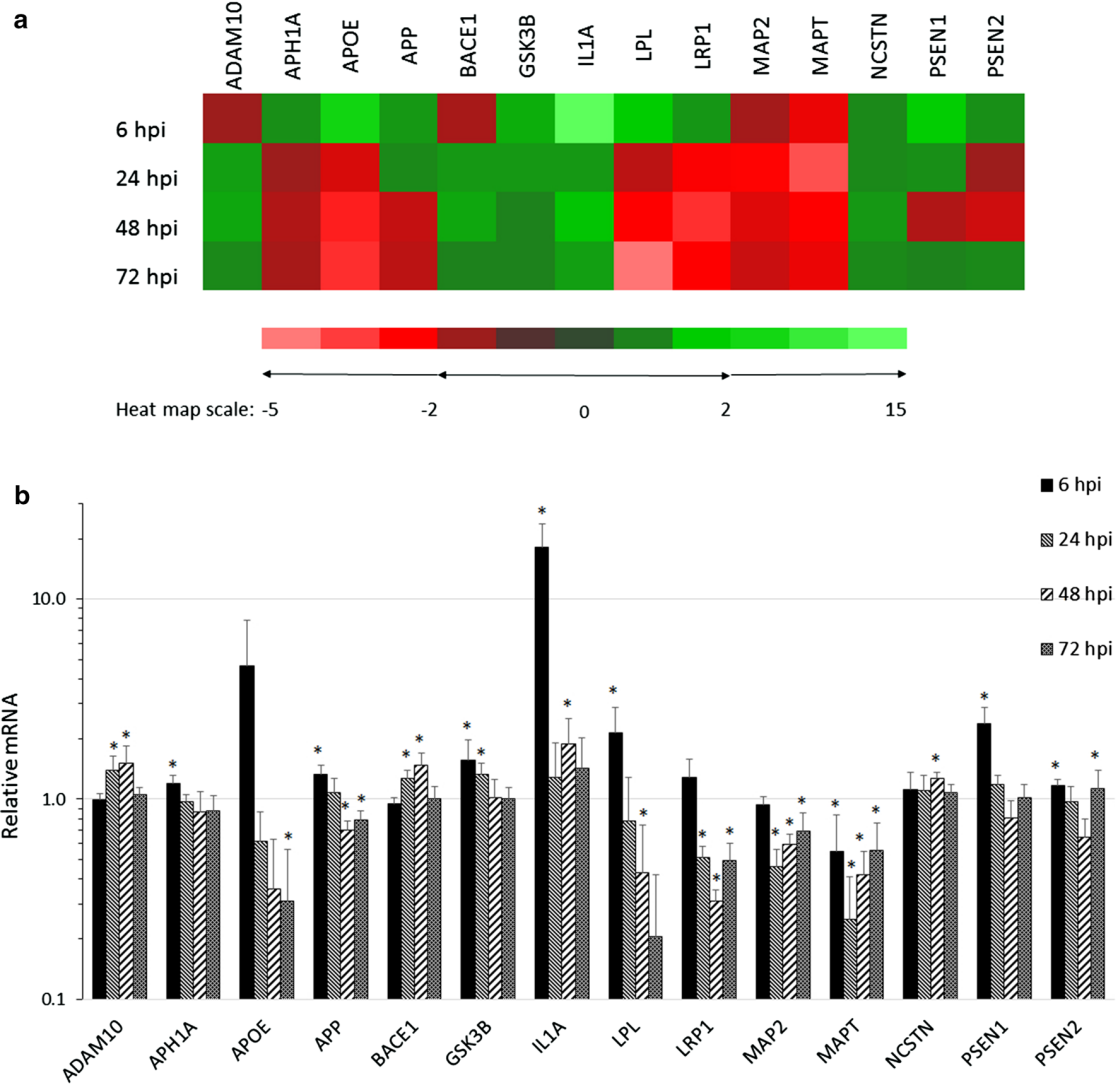
*Chlamydia pneumoniae* infection of human astrocytes alters the transcript expression of AD-related genes. Gene transcripts from *Cpn*-infected and uninfected cells analyzed at all four timepoints post-infection revealed significant fold changes in genes closely related to AD pathology. The fold changes of fourteen genes implicated in known pathways of AD pathology are presented in **a**. Histograms of fold changes of these AD-associated genes are presented in **b**. All expression data was normalized to β-actin and *Cpn*-infected and uninfected cDNA samples were repeated in biological (*N* = 3) and technical triplicate for each timepoint. Asterisk indicates *p* < 0.05. ADAM10, A disintegrin and metalloproteinase 10; APH1A, anterior pharynx defective protein 1A; APOE, apolipoprotein E; APP, amyloid precursor protein; BACE1, βAPP-cleaving enzyme 1; GSK3B, glucogen synthase kinase 3-β; IL1A, interleukin 1α; LPL, lipoprotein lipase; lipoprotein receptor-related protein 1, LRP1; MAP2, microtubule associated protein 2; MAPT, microtubule associated protein tau; NCSTN, nicastrin; PSEN1, presenilin-1, PSEN2, presenilin-2

The human Alzheimer disease array revealed that *Cpn* infection at each timepoint post-infection altered the expression of several genes directly and indirectly involved with the development of AD pathology through APP processing and tau-related mechanisms. The fold changes in expression of the 84 AD-related genes in *Cpn*-infected cells, compared to that of uninfected cells were standardized to β-actin. At 6, 24, 48, and 72 hpi, significant changes in mRNA expression were observed in 40, 33, 35, and 17 different genes, respectively. The remaining genes were not included in our analysis due to the lack of a significant difference in their expression (*p* > 0.05). The functional roles of these genes (Fig. 2a) in astrocytes are aberrant in multiple pathways of AD, including lipid metabolism (apolipoprotein E, APOE; lipoprotein lipase, LPL; lipoprotein receptor-related protein 1, LRP1), microtubule organization (microtubule-associated protein 2, MAP2; microtubule-associated protein tau, MAPT; glycogen synthase kinase 3β, GSK3B), and neuroinflammation (interleukin 1-α, IL1A) [29, 37–39]. Moreover, expression of genes associated with several of these pathways have been shown to be altered upon infection with *Cpn* [40–42]. An additional subset of genes included in this analysis encode the secretases involved in APP processing, as well as APP itself. The transcripts of APP, ADAM10, BACE1, and subunits of the γ-secretase complex (PSEN1, PSEN2 APH1A and NCSTN) were significantly upregulated (*p* < 0.05) in at least one of the 4 investigated timepoints post-infection (Fig. 2b).

*Cpn* had its greatest effect on the transcriptional expression of APP-processing secretases 6, 24, and 48 hpi. The altered gene expression observed as early as 6 hpi indicates that early *Cpn* entry into the astrocyte host may act as a trigger for the expression of genes required for the processing of APP. The increase in expression of APP (33%), PSEN1 (39%), PSEN2 (17%) and APH1A (20%) observed in infected cells compared to uninfected cells was greatest at 6 hpi, while that of ADAM10 and BACE1 was most elevated at 24 and 48 hpi (approximately 30–50% over that of uninfected cells). PSEN1, PSEN2 and APH1A function in concert with nicastrin, which demonstrated the greatest increase in expression at 48 hpi (approximately 30% over that of uninfected cells), to form the γ-secretase complex (Fig. 2b).

Our data indicate that the greatest increase in expression of secretase genes occurred within the first 48 hpi, though apparently neither the pro- nor the non-amyloidogenic pathway is favored, since expression of both the α-secretase (ADAM10) and β-secretase (BACE1) were similarly increased. These findings suggest that *Cpn* infection may be enhancing the processing of APP through transcriptional upregulation of secretase-associated genes.

### *Chlamydia pneumoniae* infection of astrocytes alters the expression of the APP processing secretases

To determine whether *Cpn*-induced transcriptional changes in ADAM10, BACE1, and PSEN1 expression were consistent at the protein level, these proteins were visualized in *Cpn*-infected cells via confocal immunofluorescence. As ADAM10, BACE1, and PSEN1 proteins mature, they are recycled between the plasma and endosomal membranes [12], however, the antibodies used to visualize them in this study were chosen to be non-selective for intracellular and plasma membrane-localized populations. For example, BACE1’s C-terminal domain was targeted, which will detect BACE as it is recycled to and from endosomes and the plasma membrane or as it is shuttled through the late endosome/lysosome pathway.

**Fig. 3:**
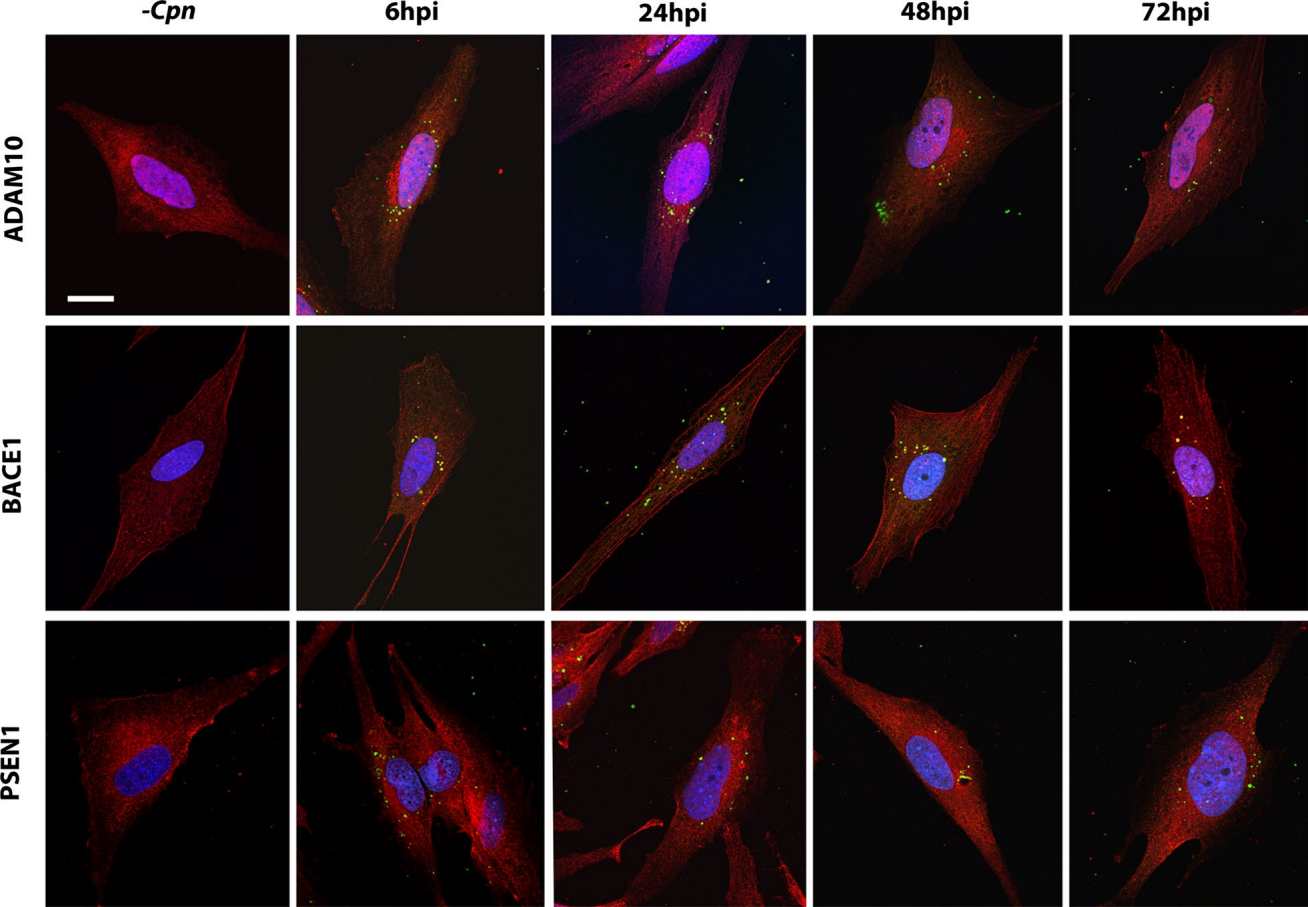
*Chlamydia pneumoniae* infection of astrocytes alters labeling of secretases. Astrocytes infected with *Cpn* from 6 to 72 hpi were double labeled for *Cpn* (green) and secretases ADAM10, BACE1 or PSEN1 C-terminal fragment (red). 10–15 cells per biological replicate were imaged against an equal number of uninfected control cells. Cells were visualized using laser scanning confocal microscopy, maintaining the voltage settings of each color channel identical across biological replicates. DAPI was used to visualize the nucleus. Representative images are included this figure. Scale bar represents 20 μm

Overall, the total fluorescence of each labeled protein in *Cpn*-infected cells showed very subtle differences at each timepoint post-infection compared to that of uninfected cells. ADAM10 labeling was not qualitatively different in *Cpn*-infected versus uninfected cells; BACE1 and PSEN1 labeling did show noticeable variations post-*Cpn* infection (Fig. 3). In our studies, the pattern of BACE1 labeling differed between uninfected and infected astrocytes, potentially representing localization to cellular membranes in *Cpn*-infected cells, though the intensity of BACE1 labeling appeared unchanged. Labeling of PSEN1, on the other hand, did appear increased in *Cpn*-infected relative to uninfected astrocytes.

### *Chlamydia pneumoniae* infection of astrocytes increases the protein expression of secretases involved in APP processing

**Fig. 4:**
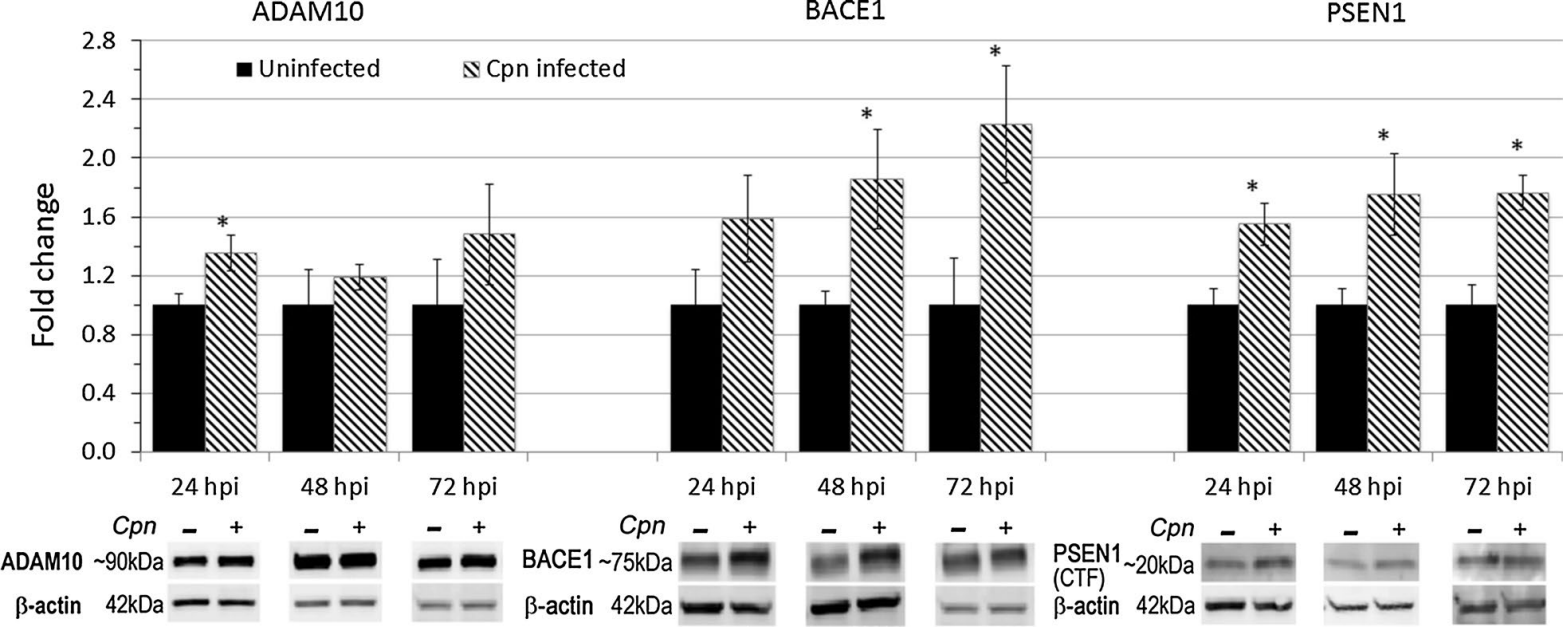
Protein expression of ADAM10, BACE1 and PSEN1 in *Chlamydia pneumoniae*-infected and uninfected astrocytes. Whole cell lysate was harvested from *Cpn*-infected and uninfected astrocytoma cells, resolved via SDS-PAGE gel electrophoresis, and labeled for secretase proteins. Fold change represents densitometry analysis for protein levels of full-length ADAM10, BACE1 and PSEN1 C-terminal fragment in *Cpn*-infected cells compared to that of uninfected cells at the same timepoint post infection. All densitometry values were normalized to that of β-actin for each biological replicate (*N* = 5–7). Statistical analysis was conducted using student’s *T*-test of fold change within each timepoint (asterisk indicates *p* < 0.05). Error bars represent standard error of the mean

To examine *Cpn*’s regulatory dynamics on the non-amyloidogenic processing pathway, total cell lysates from *Cpn* infected and uninfected cells at 24, 48, and 72 hpi were harvested for western blot analysis to semi-quantitatively assess the expression of ADAM10 (α-secretase), BACE1 (β-secretase) and PSEN1 (a component of the γ-secretase complex). Densitometry histograms of fold change of ADAM10, BACE1 or PSEN1 protein levels in *Cpn*-infected cells represent the average change in protein levels across 5–7 replicates per time point, normalized to that of β-actin within each sample and shown relative to the amount of the respective protein present in uninfected cells for each timepoint (Fig. 4).

The ADAM10 antibody used for western blots detected an immature, proenzyme form of ADAM10 (~ 90 kDa), the initiating enzyme for this pathway. Full length ADAM10 requires posttranslational modifications to be active, therefore the ADAM10 labeling presented in these analyses detects inactive enzyme [12]. Compared to that of uninfected samples, *Cpn*-infected astrocyte cell lysates showed a statistically significant 1.4 fold increase in ADAM10 protein levels (*p* < 0.05) at 24 hpi, but no significant changes were measured at other timepoints, although the trend at 72 hpi indicated increased protein levels in infected cells at this timepoint (Fig. 4).

The protein expression of BACE1 was assessed to determine *Cpn*’s effect on the pro-amyloidogenic processing of APP. The BACE1 labeling observed at approximately 75 kDa corresponds to the mature form of the protein. Mature BACE1 protein levels detected in *Cpn*-infected versus uninfected cell lysates significantly increased by 1.9 and 2.2 fold after 48 and 72 hpi, respectively, over that of uninfected cells (Fig. 4). These data suggest that *Cpn* infection results in a more extensive increase in protein levels of intracellular BACE1 relative to ADAM10, which may promote a pro-amyloidogenic rather than a non-amyloidogenic processing pathway of APP. Additionally, the increase in BACE1 protein expression observed with these studies does not seem transient; as time of infection progresses, BACE1 protein levels continued to increase throughout the 72 h of infection.

Active PSEN1 is localized to numerous subcellular compartments of the cell [43]. The antibody used for PSEN1 labeling via western blot analysis did not detect intracellular levels of full length PSEN1, which appears at 50 kDa, but rather a 20 kDa band, indicating proteolytically cleaved, and therefore active, carboxy terminal fragment (CTF) of PSEN1. At all timepoints tested (24, 48, and 72 hpi), PSEN1 CTF protein levels detected in *Cpn*-infected cell lysates were significantly increased over those of uninfected controls (*p* < 0.05) (Fig. 4). Corroborating the observations made via confocal immunofluorescence, these western blot results provide additional evidence for the role of *Cpn* in facilitating the accumulation of potentially active PSEN1.

### Fluorescence intensity of Aβ_1-42_ is increased in *Chlamydia pneumoniae* infected astrocytes

**Fig. 5:**
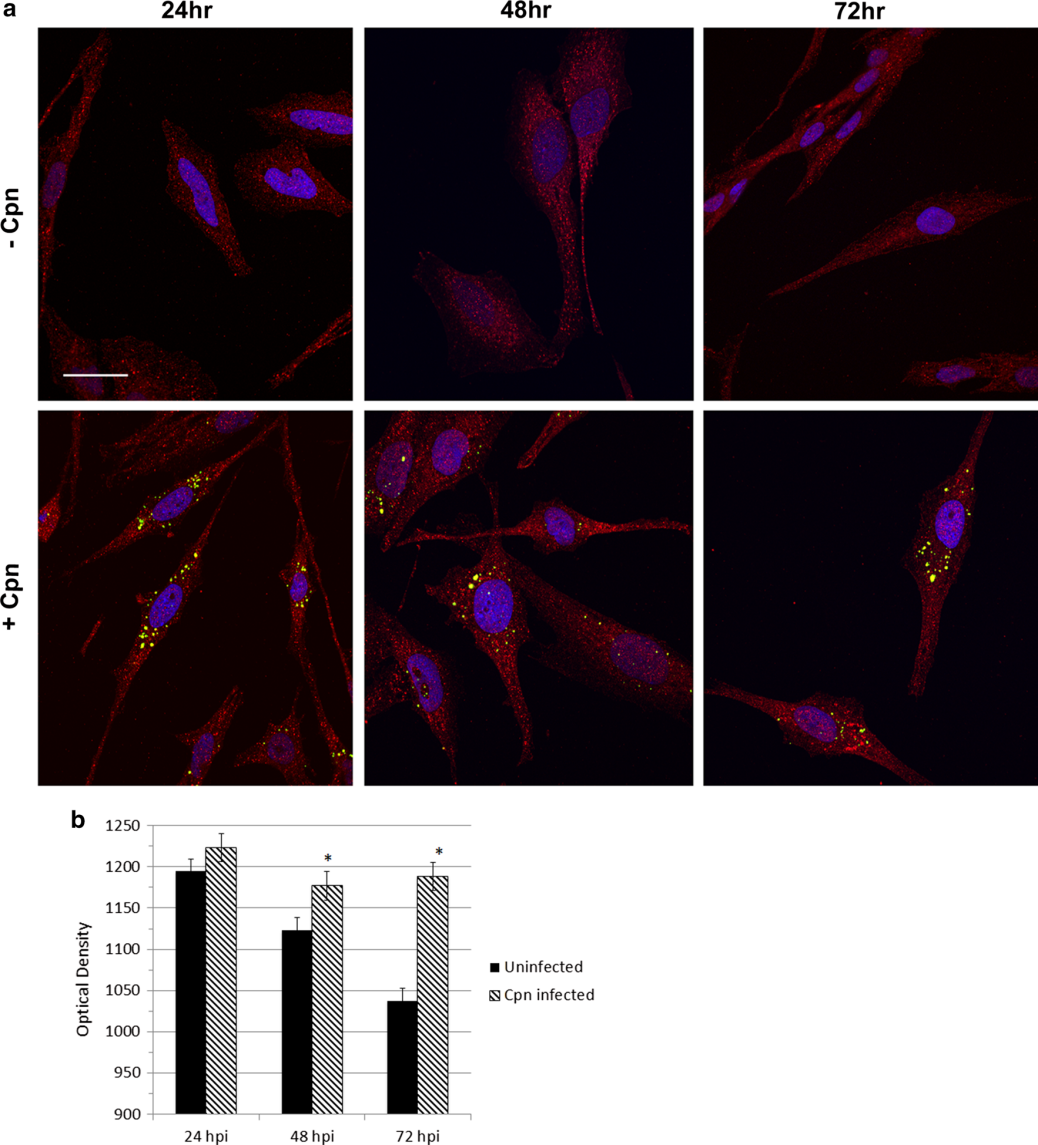
Fluorescence intensity of Aβ_1-42_ is increased in *Chlamydia pneumoniae*-infected astrocytes. Aβ_1-42_ (red) and *Cpn* (green) were visualized by laser scanning, confocal microscopy (**a**). To analyze the Z-images using FIJI software, a defined threshold subtraction was applied equally to each image to determine Aβ_1-42_ fluorescence intensity (**b**); mean fluorescence intensity was calculated for infected and uninfected astrocytes at 24, 48 and 72 hpi. Cells (*N* = 25–30) were analyzed across three biological replicates to reliably conduct a student’s *t*-test on the fluorescence intensities of Aβ_1-2_ in uninfected and *Cpn*-infected cells. Error bars represent standard error of the mean. Asterisk represents *p* < 0.05

Approximately 30 Z-images of uninfected and *Cpn*-infected cells were acquired for each timepoint and resolved into a single, 2D representation of the maximum Aβ_1-42_ fluorescence intensity of each 0.2 μm optical section (Fig. 5a). Aβ_1-42_ was localized within all cells, regardless of treatment group, indicating constitutively active APP-processing. Mean Aβ_1-42_ fluorescence intensity in *Cpn*-infected astrocytes, relative to that of uninfected cells, did not differ significantly at 24 hpi; however, it was elevated in infected cells at 48 and 72 hpi relative to uninfected cells from the same timepoint (Fig. 5b), indicating an increase in pro-amyloidogenic processing of APP at later times of infection.

### *Chlamydia pneumoniae* infection alters the activity of ADAM10 and BACE1 in initiating cleavage of APP

**Fig. 6:**
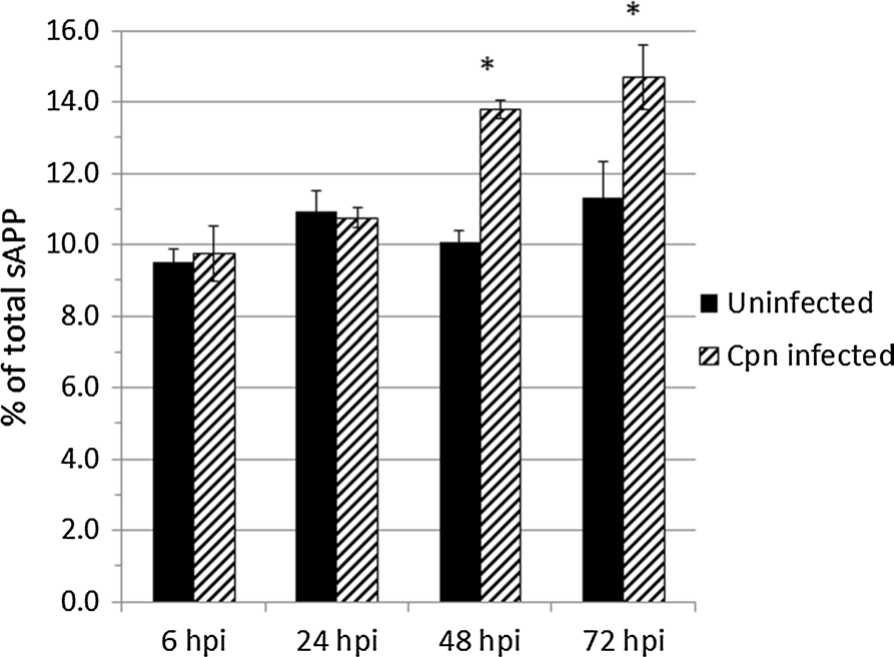
Quantification of sAPPβ/total sAPP in media of uninfected and *Chlamydia pneumoniae* infected astrocytes using MSD ELISA. Conditioned media of uninfected and *Cpn*-infected cells at each timepoint post-infection was collected, concentrated, and assayed in equal volumes for sAPPβ and sAPPα levels. Standard curves of known concentrations of sAPPβ and sAPPα was used to determine the concentration of these individual Aβ species. Conditioned media was obtained from three biological replicates and the assay was conducted in technical triplicate. Student’s *t*-test was calculated using the average sAPPβ/total sAPP ratio of *Cpn*-infected conditioned media compared to that of uninfected conditioned media. Asterisk represents *p* < 0.05

To study *Cpn*’s effect on ADAM10 and BACE1 activities in the initial cleavage of APP, MSD ELISA was used to quantify the concentration of soluble APPα (sAPPα) and soluble APPβ (sAPPβ) in the conditioned media of uninfected and *Cpn*-infected cells. Corroborating the significantly increased intracellular Aβ_1-42_ at 48 and 72 hpi, we observed a significant increase in the ratio of sAPPβ/total sAPP, expressed as a percentage (Fig. 6), at 48 and 72 hpi in *Cpn*-infected cells when compared to that of uninfected cells (*p* < 0.05). The levels of sAPPβ relative to total sAPP released by uninfected cells did not vary significantly between time points, suggesting a regulated balance of APP processing by ADAM10 and BACE1.

### *Chlamydia pneumoniae* infection of astrocytes results in increased activity of BACE1 and decreased activity of ADAM10

**Fig. 7:**
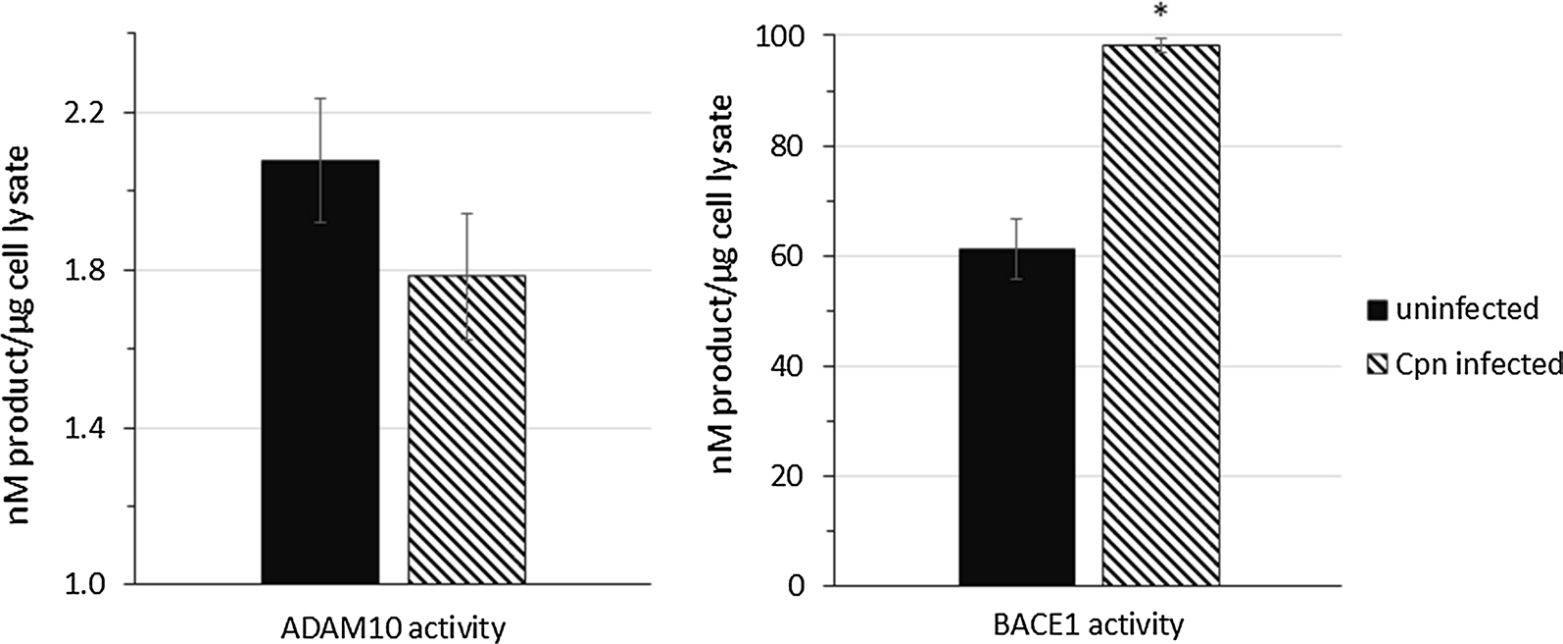
BACE1 activity is increased and ADAM10 activity is decreased in *Chlamydia pneumoniae* infected astrocytes. FRET-based assays were used to quantify the activity of ADAM10 and BACE1 enzyme activity generated by *Cpn*-infected and uninfected cell lysates at 48 hpi. Fluorescence of the 5-FAM or HiLyte Fluor 488 dyes conjugated to ADAM10 or BACE1-specific substrates was detected as a result of cleavage by the respective enzymes and compared back to fluorescence intensity of known dye concentrations. The quantified dye concentration from ADAM10 and BACE1 fluorescent substrate cleavage from (*N* = 4) biological replicates of *Cpn*-infected and uninfected cell lysates is presented as an average in the histograms. Error bars represent standard error of the mean. Asterisk represents *p* < 0.05

The enzymatic activity of ADAM10 and BACE1 is contingent upon post-translational modifications that affect each enzyme’s trafficking to the optimal subcellular compartments for APP cleavage [12, 44]. To determine if *Cpn*-induced altered expression of ADAM10 and BACE1 at the protein level correlated to their altered enzymatic activity, fluorimetric FRET-based enzyme assays were conducted on whole cell lysate of uninfected and *Cpn*-infected cells. Compared to that of uninfected cells, the concentration of ADAM10-cleaved, fluorescent substrate in *Cpn*-infected cell lysate at 48 hpi exhibited an overall decreased trend in ADAM10 activity (Fig. 7). In contrast, the concentration of BACE1-cleaved, fluorescent substrate generated by *Cpn*-infected cell lysate 48 hpi was significantly greater than that of uninfected cell lysate (*p* < 0.05), indicating an overall greater activity of BACE1 in these samples (Fig. 7).

## Discussion

By investigating *Cpn*’s effect on the transcription and localization of APP-processing secretases, this study is the first to propose a mechanism by which a respiratory strain of *Cpn* alters APP processing in an astrocyte host. Balin et al. [23] identified the *Cpn* pathogen within neurons, microglia and astroglia of AD brain tissue. Following this initial study, numerous groups have explored the roles of astrocytes as hosts for *Cpn* [45–47]. While our data indicate that *Cpn* clearly infects astrocytes, the percent of astrocytes that were infected decreased over time. The life cycle of *Cpn* is complex and involves transitioning from an infectious, elementary body (EB) to a metabolically active, reticulate body (RB) form of growth before converting back to an EB that can be released to infect new cells. Under conditions of stress, *Cpn* can persist as viable but noninfectious aberrant bodies (AB), which can revert back to RBs when conditions favor active multiplication [48]. It is possible that if cell division occurs more rapidly than *Cpn* can complete its life cycle and spread to a new host cell, then the number of infected cells relative to uninfected cells will decrease over time. Alternatively, it is possible that *Cpn* forms ABs in astrocytes, which would not subsequently spread to astrocytes arising from division of uninfected cells. This also could account for the decrease in % infected astrocytes observed over time.

In this study, we report that having gained access into the astrocyte host, *Cpn* promotes significant dysregulation of important AD-related genes directly involved with APP processing, pathologic lipid trafficking and microtubule dysfunction. While physiologically abnormal and potentially harmful for a mammalian host, the altered expression of these genes may have distinct benefits for *Cpn* infecting target cells. For example, *Cpn* infection of endothelial cells, monocytes, and macrophages has been shown to alter the expression of lipid homeostasis genes [40, 42, 49] and allow the acquisition and utilization of host lipids by *Cpn* as the pathogen is unable to synthesize them de novo [50]. The increased mRNA levels of apolipoprotien E (ApoE) and lipoprotein lipase (LPL) observed in *Cpn*-infected astrocytes relative to uninfected cells at 6 hpi may therefore be necessary to allow *Cpn* to initiate infection within the astrocyte host. In addition, APOE has been shown to play a role in the attachment and internalization of several intracellular pathogens, including *Cpn* [51–53]. The observed early increase in ApoE transcript expression may enhance internalization of *Cpn* EB’s during infection. *Cpn*-induced changes in host gene expression presumably evolved to enhance the infectivity of the bacterium, while potential AD-related pathologic effects associated with altered host gene expression would be an indirect consequence of *Cpn* infection.

It is conceivable that the initial increase in transcriptional ApoE expression has a direct impact on expression of APP. A recent study noted that the binding of glial ApoE to its receptors enhances the transcription of APP through activating the transcription factor activator protein-1 (AP-1) and its associated family of downstream effectors [54], the consequence of which may account for the 30% increase in APP transcription seen at 6 hpi. Furthermore, infection of endothelial cells by *Cpn* activates AP-1, and activation of this transcription factor regulates *Cpn*-induced inflammation [55]. If a similar scenario occurs in *Cpn*-infected astrocytes, AP-1 may play a role in mediating *Cpn*-triggered neuroinflammation as well as modulating levels of APP, and indirectly, Aβ levels. Data presented here indicate that *Cpn* stimulated transcription of the proinflammatory cytokine interleukin 1α (IL1α), suggesting that infection of astrocytes by *Cpn* promoted an inflammatory response. Moreover, a study by Lim et al. [41] reported that *Cpn* can activate an inflammatory response in monocytes, which, if occurring in microglial cells of the central nervous system, would reactivate nearby astrocytes. It is likely that *Cpn* infection in the human brain would trigger an inflammatory response that would exacerbate neurodegeneration associated with AD.

In this study, it is shown that *Cpn* infection of astrocytes decreased levels of microtubule-associated protein tau (MAPT) and microtubule-associated protein 2 (MAP2) mRNA, and increased that of glycogen synthase kinase 3-β (GSK3β), a kinase that can phosphorylate tau. A decrease in tau protein would destabilize host microtubules, whereas an increase in tau phosphorylation would subsequently decrease tau binding to, and stabilization of, microtubules [56]. An increase in GSK3β activity in *Cpn*-infected astrocytes could potentially enhance the formation of neurofibrillary tangles, which are composed of hyperphosphorylated tau proteins, thereby contributing to tau-mediated pathology that occurs in AD.

Changes in the expression of ADAM10, BACE1, and PSEN1 in *Cpn*-infected astrocytes will directly affect the processing of APP. In the present study, we observed significant increases in the expression of ADAM10 mRNA by 40–50% in *Cpn*-infected astrocytes relative to uninfected cells, as well an increase in the full length ADAM10 protein. However, it is important to note that the ADAM10 proenzyme needs posttranslational processing to be active and thus protein levels determined in this study may not accurately represent enzymatically active ADAM10 [7, 12]. In fact, ADAM10 protein levels tended to increase with *Cpn* infection while enzymatic activity was diminished at 48 hpi. These data suggest that posttranslational modification and/or trafficking of ADAM10 may differ in infected and uninfected astrocytes, resulting in decreased α-secretase-mediated non-amyloidogenic cleavage of APP in *Cpn*-infected cells.

Protein levels of both BACE1 and PSEN1 in *Cpn*-infected astrocytes progressively increased from 24 to 72 hpi relative to uninfected cells. Relative mRNA levels were greatest at 48 hpi for BACE1 and 6 hpi for PSEN1, indicating that the proteins persisted after transcription of these genes returned to levels consistent with that seen in uninfected astrocytes. The progressive increase in the amount of BACE1 and PSEN1 in *Cpn*-infected astrocytes over time paralleled the observation that Aβ_1-42_ labeling was greatest in infected cells at 48 and 72 hpi. It has been shown that in the presence of Aβ_1-42_, BACE1 activity is elevated because of impaired lysosomal degradation of BACE1, indicating that increased pro-amyloidogenic processing of APP favors persistence of this β-secretase [57, 58]. This positive feedback between Aβ_1-42_ and BACE1 levels may have occurred in our *Cpn*-infected astrocytes. Furthermore, it has been shown that low-density lipoprotein receptor-related protein 1 (LRP1) regulates BACE1 expression and activity by directing the β-secretase to lysosomes for degradation, while a loss of LRP1 expression correlates with an increase in BACE1 activity [59]. Thus the decreased transcription of LRP1 observed in *Cpn*-infected astrocytes may contribute to the increase in BACE1 expression and activity observed in infected astrocytes.

The lack of a significant increase in the fluorescence intensity of Aβ_1-42_ at 24 hpi suggests that prior to 24 hpi, the pro-amyloidogenic pathway has not been upregulated. If the increase in APP mRNA observed in *Cpn*-infected astrocytes at 6 hpi signals an increase in APP protein, then the surplus APP within the cell is processed by the non-amyloidogenic pathway at an early time of infection. However, at 48 to 72 hpi, the significant increase in Aβ_1-42_ fluorescence labeling in infected astrocytes suggests that in the presence of *Cpn*, either pro-amyloidogenic processing of APP is stimulated and/or there is decreased clearance of toxic, intracellular Aβ products. The increased protein concentration of BACE1 and PSEN1 present in infected astrocytes at 48 and 72 hpi supports the conclusion that increased processing of APP may be responsible for the greater intracellular accumulation of Aβ_1-42_, however doesn’t rule out the possibility of decreased clearance of Aβ. Our data indicating that the ratio of sAPPβ per total sAPP (sAPPα and sAPPβ) was significantly increased in *Cpn*-infected astrocytes at 48 and 72 hpi further supports the conclusion that APP processing favored the pro-amyloidogenic pathway as the infection progressed. These results could be explained by either an increase in BACE1-mediated cleavage and/or a decrease in ADAM10-mediated cleavage of APP. Interestingly, enzyme activity assays for BACE1 and ADAM10 indicated that at 48 hpi, enzymatic activity in *Cpn*-infected astrocytes was increased for BACE1 and decreased for ADAM10 relative to that measured in uninfected cells.

Our study supports the postulate that the mechanism by which *Cpn* induces AD pathology centers on the ability of the pathogen in astrocytes to temporally alter the expression and activity of the α- and β-secretases, and thereby alter the balance between the non- and pro-amyloidogenic APP processing pathways that occurs in uninfected cells. This study is the first to quantify the altered regulation of the predominant α, β-, and γ-secretases in the CNS, namely ADAM10, BACE1, and PSEN1, respectively, and Aβ_1-42_ in human astrocytes infected with *Cpn*. We have demonstrated that *Cpn* infection causes a significant increase in the amyloidogenic processing of APP, which correlates with increased protein levels and activity of the rate-limiting enzyme, BACE1. Whether through upregulating the transcriptional or post-transcriptional expression of BACE1 and the subunits of γ-secretase and/or simultaneously disrupting normal secretase trafficking, severe downstream effects on the CNS may result secondary to *Cpn* infection. Over time, *Cpn*-induced astrocyte activation culminating in neuroinflammation, altered APP processing favoring the amyloidogenic pathway, dysregulation of tau expression and function, and eventually neuronal death causes chronic, irreversible damage, resulting in pathology similar to that found in the CNS of AD patients.

## Future directions

This investigation explores a potential *Cpn*-induced mechanism for Aβ formation by focusing on the pathway of APP processing by proamyloidogenic secretases, thereby identifying a putative early event triggering AD-associated pathology. While this study is the first to model a stable, *Chlamydia pneumoniae*-infection of human astrocytes in vitro and investigate its downstream effects on AD-related secretases, numerous additional studies can be conducted to further support our conclusions. The STTG1 human astrocytoma cell line has been used as a viable astrocyte model for AD [31–34]. Considering *Cpn* has been found in multiple areas of human cortex and vasculature as reported by Balin et al. [23], reproducing *Cpn* infection in additional cell types such as other astrocyte and glial cell lines, neuronal cells, and endothelial cells may provide a more thorough understanding of in vivo *Cpn* infection. Reversing the pathologic effects of Cpn infection with anti-microbial or anti-inflammatory medication may provide a viable therapeutic option for AD. In support of this, Hammond et al. [60] demonstrated decreased cerebral Aβ load in BALB/c mice inoculated with *Cpn* and subsequently treated with Moxifloxacin. Recent studies implicating pathogens in AD, including this current study, suggest that eliminating infectious triggers for AD pathology may be beneficial as a therapeutic target for preventing the initiation or progression of AD (Additional file 2).

## Conclusions

This study examines the effect of *Chlamydia pneumoniae* infection on astrocytes, a major cell type in the CNS that plays an important role in establishing a state of neuroinflammation and neurodegeneration in the brain. Specifically, the data suggest that infection by *Chlamydia pneumoniae* promotes the pro-amyloidogenic pathway of APP processing by manipulating the expression and activity of the major secretases involved in generating toxic and nontoxic fragments of APP. Pro-inflammatory processes in the brain, and environmental stimuli that favor the pro-amyloidogenic pathway of APP processing, are emerging as potential triggers for the pathology associated with AD. We present evidence of an association between AD pathology and infection with *Chlamydia pneumoniae*, supporting the concept of an infectious etiology as a candidate to be considered in the pathogenesis of late onset AD. Furthermore, this study presents a potential target for preventing or slowing the progression of this neurodegenerative disease.

## Additional files

**Additional file 1: AD array tables.** Table S1. AD-associated genes whose expression was significantly altered in *Cpn*-infected cells relative to uninfected cells at 6 hpi. Table S2. AD-associated genes whose expression was significantly altered in *Cpn*-infected cells relative to uninfected cells at 24 hpi. Table S3. AD-associated genes whose expression was significantly altered in *Cpn*-infected cells relative to uninfected cells at 48 hpi. Table S4. AD-associated genes whose expression was significantly altered in *Cpn*-infected cells relative to uninfected cells at 72 hpi. Description: These tables list the Alzheimer-associated genes whose expression in *Cpn*-infected cells significantly differed from that of uninfected cells. The genes examined were included in the Human Alzheimer Disease RT^2^ Profiler ™ PCR Array (Qiagen, PAHS-057ZC) that profiled 84 different Alzheimer-associated genes.

**Additional file 2: MSD ELISA %CV values.** Table S1. Percent CV for soluble APPα. Table S2. Percent CV for soluble APPβ. Description: These tables list intra- and inter-ELISA % coefficient of variation (CV) for the data generated by MSD ELISA.

## Abbreviations

Aβ: beta-amyloid
AD: Alzheimer disease
ADAM10: a disintegrin and metalloproteinase-10
AICD: APP intracellular domain
ANOVA: analysis of variance
AP-1: activator protein-1
APH-1: anterior pharynx defective 1
ApoE: apolipoprotein E
APP: amyloid precursor protein
BACE1: β-site APP cleaving enzyme 1
BCA: bicinchoninic acid
cDNA: complementary deoxyribonucleic acid
CNS: central nervous system
*Cpn*: *Chlamydia pneumoniae*
CTF: carboxy-terminal fragment
DAPI: 4′,6-diamidino-2-phenylindole
EB: elementary body
EOAD: early onset Alzheimer disease
ER: endoplasmic reticulum
FAD: familial Alzheimer disease
FBS: fetal bovine serum
FITC: fluorescein isothiocyanate
FRET: fluorescence resonance energy transfer
GFAP: glial fibrillary acidic protein
GSK3β: glycogen synthase kinase 3-β
hpi: hours post-infection
IFN-y: Interferon-y
IL-1α: interleukin 1-α
IL-1β: interleukin 1-β
LOAD: late onset Alzheimer disease
LPL: lipoprotein lipase
LRP1: lipoprotein receptor-related protein-1
MAP2: microtubule associated protein 2
MAPT: microtubule associated protein tau
mRNA: messenger RNA
MSD: meso scale discovery
ELISA: enzyme-linked immunosorbent assay
NCSTN: nicastrin
PBS: phosphate buffered saline
PCR: polymerase chain reaction
PEN2: presenilin enhancer-2
PET: positron emission tomography
PSEN1: presenilin-1
PSEN2: presenilin-2
RB: reticulate body
RFU: relative fluorescence unit
RT-PCR: real time polymerase chain reaction
SAD: sporadic Alzheimer disease
sAPPα: soluble APPα
sAPPβ: soluble APPβ
SDS-PAGE: sodium dodecyl sulphate polyacrylamide gel electrophoresis
TGN: *trans*-Golgi Network
TNF-α: tumor necrosis factor-α
TRITC: tetramethylrhodamine
Tukey’s HSD: Tukey’s honest significance difference
