# Supplementary material for: Astrocytes infected with Chlamydia pneumoniae demonstrate altered expression and activity of secretases involved in the generation of β-amyloid found in Alzheimer disease
Source: BMC Neurosci. 2019 Feb 20;20:6. doi: 10.1186/s12868-019-0489-5 (PMC6383264; doi:10.1186/s12868-019-0489-5)
Supplement: Supplementary file 1 — Additional file 1: AD array tables. Table S1. AD-associated genes whose expression was significantly altered in Cpn-infected cells relative to uninfected cells at 6 hpi. Table S2. AD-associated genes whose expression was significantly altered in Cpn-infected cells relative to uninfected cells at 24 hpi. Table S3. AD-associated genes whose expression was significantly altered in Cpn-infected cells relative to uninfected cells at 48 hpi. Table S4. AD-associated genes whose expression was significantly altered in Cpn-infected cells relative to uninfected cells at 72 hpi. Description: These tables list the Alzheimer-associated genes whose expression in Cpn-infected cells significantly differed from that of uninfected cells. The genes examined were included in the Human Alzheimer Disease RT2 Profiler ™ PCR Array (Qiagen, PAHS-057ZC) that profiled 84 different Alzheimer-associated genes. [file 12868_2019_489_MOESM1_ESM.pdf]

Table 1. AD-associated genes whose expression was significantly altered in *Cpn*-infected cells relative to uninfected cells at 6 hpi

Gene symbol	Gene description	Fold regulation	p-value
ABCA1	ATP-binding cassette, sub-family A (ABC1), member 1	2.69	<0.001
ACHE	Acetylcholinesterase	4.02	0.016
APBA1	Amyloid beta (A4) precursor protein-binding, family A, member 1	-6.69	0.030
APBA3	Amyloid beta (A4) precursor protein-binding, family A, member 3	1.45	0.006
APH1A	Anterior pharynx defective 1 homolog A (C. elegans)	1.20	0.023
APLP1	Amyloid beta (A4) precursor-like protein 1	1.58	0.006
APP	Amyloid beta (A4) precursor protein	1.33	0.010
CASP3	Caspase 3, apoptosis-related cysteine peptidase	1.64	0.008
CASP4	Caspase 4, apoptosis-related cysteine peptidase	2.30	<0.001
CDK1	Cyclin-dependent kinase 1	-1.62	0.047
CLU	Clusterin	1.65	0.027
CTSC	Cathepsin C	1.42	0.021
CTSG	Cathepsin G	1.48	0.025
CTSL	Cathepsin L1	1.45	0.001
EP300	E1A binding protein p300	1.47	0.044
ERN1	Endoplasmic reticulum to nucleus signaling 1	1.95	0.040
GNAO1	Guanine nucleotide binding protein (G protein), alpha activating activity polypeptide O	-1.83	0.014
GNAZ	Guanine nucleotide binding protein (G protein), alpha z polypeptide	-1.78	0.012
GNB1	Guanine nucleotide binding protein (G protein), beta polypeptide 1	1.52	0.013
GNG11	Guanine nucleotide binding protein (G protein), gamma 11	1.89	0.017
GNG4	Guanine nucleotide binding protein (G protein), gamma 4	22.52	0.001
GSK3A	Glycogen synthase kinase 3 alpha	1.29	0.006
GSK3B	Glycogen synthase kinase 3 beta	1.56	0.042
HSD17B10	Hydroxysteroid (17-beta) dehydrogenase 10	1.21	0.015
IDE	Insulin-degrading enzyme	-1.16	0.017
IL1A	Interleukin 1, alpha	18.12	0.001
INSR	Insulin receptor	-1.53	0.006

LPL	Lipoprotein lipase	2.14	0.013
LRP8	Low density lipoprotein receptor-related protein 8, apolipoprotein e receptor	-1.70	0.010
MAPT	Microtubule-associated protein tau	-1.82	0.042
PKP4	Plakophilin 4	-2.02	0.005
PLAT	Plasminogen activator, tissue	2.56	0.050
PLAU	Plasminogen activator, urokinase	2.47	0.000
PRKCG	Protein kinase C, gamma	3.80	0.021
PRKCI	Protein kinase C, iota	1.26	0.014
PRKCQ	Protein kinase C, theta	-1.57	0.009
PSEN1	Presenilin 1	2.39	0.005
PSEN2	Presenilin 2 (Alzheimer disease 4)	1.17	0.009
SERPINA3	Serpin peptidase inhibitor, clade A (alpha-1 antiproteinase, antitrypsin), member 3	27.73	0.010
UBQLN1	Ubiquilin 1	1.33	0.003

Table 2. AD-associated genes whose expression was significantly altered in *Cpn*-infected cells relative to uninfected cells at 24 hpi

Gene symbol	Gene description	Fold regulation	p-value
A2M	Alpha-2-macroglobulin	-2.92	0.005
ACHE	Acetylcholinesterase	2.53	0.030
ADAM10	ADAM metallopeptidase domain 10	1.40	0.013
APBA1	Amyloid beta (A4) precursor protein-binding, family A, member 1	-5.48	0.004
APOA1	Apolipoprotein A-I	-1.70	0.006
BACE1	Beta-site APP-cleaving enzyme 1	1.27	0.013
BDNF	Brain-derived neurotrophic factor	2.16	0.004
CDK1	Cyclin-dependent kinase 1	1.17	0.012
CTSB	Cathepsin B	-1.27	0.010
CTSL	Cathepsin L1	-1.13	0.036
EP300	E1A binding protein p300	1.41	0.013
GNAO1	Guanine nucleotide binding protein (G protein), alpha activating activity polypeptide O	-2.30	0.008
GNAZ	Guanine nucleotide binding protein (G protein), alpha z polypeptide	-1.55	0.036
GNB1	Guanine nucleotide binding protein (G protein), beta polypeptide 1	1.23	0.014
GNG11	Guanine nucleotide binding protein (G protein), gamma 11	3.19	<.001
GNG3	Guanine nucleotide binding protein (G protein), gamma 3	-1.25	0.007
GNG4	Guanine nucleotide binding protein (G protein), gamma 4	3.42	0.004
GNGT1	Guanine nucleotide binding protein (G protein), gamma transducing activity polypeptide 1	4.91	0.001
GSK3B	Glycogen synthase kinase 3 beta	1.33	0.016
LRP1	Low density lipoprotein receptor-related protein 1	-1.95	0.001
MAP2	Microtubule-associated protein 2	-2.17	0.008
MAPT	Microtubule-associated protein tau	-3.98	0.033
NAE1	NEDD8 activating enzyme E1 subunit 1	1.42	0.002
NTRK1	Neurotrophic tyrosine kinase, receptor, type 1	-17.81	0.006
PLAT	Plasminogen activator, tissue	1.29	0.044
PLAU	Plasminogen activator, urokinase	9.57	<0.001

PRKCD	Protein kinase C, delta	-2.06	0.002
PRKCE	Protein kinase C, epsilon	-1.46	0.027
PRKCQ	Protein kinase C, theta	-2.58	<0.001
SERPINA3	Serpin peptidase inhibitor, clade A (alpha-1 antiproteinase, antitrypsin), member 3	2.77	0.034
UBQLN1	Ubiquilin 1	1.81	<0.001
UQCRC1	Ubiquinol-cytochrome c reductase core protein I	1.37	0.011
UQCRC2	Ubiquinol-cytochrome c reductase core protein II	1.21	0.033

Table 3. AD-associated genes whose expression was significantly altered in *Cpn*-infected cells relative to uninfected cells at 48 hpi

Gene symbol	Gene description	Fold regulation	p-value
A2M	Alpha-2-macroglobulin	-1.83	0.008
ABCA1	ATP-binding cassette, sub-family A (ABC1), member 1	-3.31	<0.001
ADAM10	ADAM metallopeptidase domain 10	1.52	0.025
APP	Amyloid beta (A4) precursor protein	-1.43	0.002
BACE1	Beta-site APP-cleaving enzyme 1	1.47	0.004
CDK1	Cyclin-dependent kinase 1	2.31	<0.001
CLU	Clusterin	-1.65	0.002
CTSC	Cathepsin C	1.24	0.043
CTSD	Cathepsin D	-1.70	0.002
CTSL	Cathepsin L1	-1.51	0.001
ERN1	Endoplasmic reticulum to nucleus signaling 1	-3.41	<0.001
GNAO1	Guanine nucleotide binding protein (G protein), alpha activating activity polypeptide O	-3.41	<0.001
GNB1	Guanine nucleotide binding protein (G protein), beta polypeptide 1	1.41	0.031
GNB5	Guanine nucleotide binding protein (G protein), beta 5	-1.23	0.033
GNG11	Guanine nucleotide binding protein (G protein), gamma 11	4.14	<0.001
GNG4	Guanine nucleotide binding protein (G protein), gamma 4	1.90	0.022
GNGT1	Guanine nucleotide binding protein (G protein), gamma transducing activity polypeptide 1	14.20	0.009
IL1A	Interleukin 1, alpha	1.89	0.044
INSR	Insulin receptor	-1.74	0.002
LPL	Lipoprotein lipase	-2.34	0.043
LRP1	Low density lipoprotein receptor-related protein 1	-3.22	<0.001
LRP8	Low density lipoprotein receptor-related protein 8, apolipoprotein e receptor	1.41	0.019
MAP2	Microtubule-associated protein 2	-1.68	0.001
MAPT	Microtubule-associated protein tau	-2.38	0.002
NCSTN	Nicastrin	1.27	0.004
NTRK1	Neurotrophic tyrosine kinase, receptor, type 1	-29.46	<0.001

PKP4	Plakophilin 4	1.54	0.001
PLAT	Plasminogen activator, tissue	1.18	0.030
PLAU	Plasminogen activator, urokinase	2.11	0.008
PRKCD	Protein kinase C, delta	-1.50	0.033
PRKCE	Protein kinase C, epsilon	-1.71	0.045
PRKCQ	Protein kinase C, theta	-1.86	0.023
PSEN2	Presenilin 2 (Alzheimer disease 4)	-1.55	0.029
UBQLN1	Ubiquilin 1	1.23	0.044
UQCRC1	Ubiquinol-cytochrome c reductase core protein I	1.32	0.022

Table 4. AD-associated genes whose expression was significantly altered in *Cpn*-infected cells relative to uninfected cells at 72 hpi

Gene symbol	Gene description	Fold regulation	p-value
A2M	Alpha-2-macroglobulin	-2.44	0.003
APLP1	Amyloid beta (A4) precursor-like protein 1	-2.76	0.001
APOE	Apolipoprotein E	-3.21	0.042
APP	Amyloid beta (A4) precursor protein	-1.27	0.010
CLU	Clusterin	-1.60	0.002
GNAO1	Guanine nucleotide binding protein (G protein), alpha activating activity polypeptide O	-1.72	0.010
GNG11	Guanine nucleotide binding protein (G protein), gamma 11	1.82	<0.001
LRP1	Low density lipoprotein receptor-related protein 1	-2.03	0.006
MAP2	Microtubule-associated protein 2	-1.45	0.028
MAPT	Microtubule-associated protein tau	-1.81	0.048
NAE1	NEDD8 activating enzyme E1 subunit 1	1.26	0.028
NTRK1	Neurotrophic tyrosine kinase, receptor, type 1	-19.37	0.012
PLAU	Plasminogen activator, urokinase	2.54	0.005
PRKCQ	Protein kinase C, theta	-2.20	0.025
PRKCZ	Protein kinase C, zeta	-2.81	0.014
SNCA	Synuclein, alpha (non A4 component of amyloid precursor)	1.62	0.004
SNCB	Synuclein, beta	1.79	0.002
