# Supplementary material for: Astrocytes infected with Chlamydia pneumoniae demonstrate altered expression and activity of secretases involved in the generation of β-amyloid found in Alzheimer disease
Source: BMC Neurosci. 2019 Feb 20;20:6. doi: 10.1186/s12868-019-0489-5 (PMC6383264; doi:10.1186/s12868-019-0489-5)
Supplement: Supplementary file 2 — Additional file 2: MSD ELISA %CV values. Table S1. Percent CV for soluble APPα. Table S2. Percent CV for soluble APPβ. Description: These tables list intra- and inter-ELISA % coefficient of variation (CV) for the data generated by MSD ELISA. [file 12868_2019_489_MOESM2_ESM.pdf]

MSD ELISA %CV Values

Table 1: %CV for soluble APPα

		Intra-Assay CV %	Inter-Assay CV %
**6 hpi. UN**	**Biological Replicate 1**	0.352	5.59
	**Biological Replicate 2**	0.547	
	**Biological Replicate 3**	2.06	
**6 hpi. *Cpn***	**Biological Replicate 1**	4.08	9.86
	**Biological Replicate 2**	1.32	
	**Biological Replicate 3**	4.13	
**24 hpi. UN**	**Biological Replicate 1**	2.78	10.86
	**Biological Replicate 2**	0.916	
	**Biological Replicate 3**	1.55	
**24 hpi. *Cpn***	**Biological Replicate 1**	2.42	4.67
	**Biological Replicate 2**	4.83	
	**Biological Replicate 3**	2.42	

**48 hpi. UN**	**Biological Replicate 1**	7.58	75.38
	**Biological Replicate 2**	6.2	
	**Biological Replicate 3**	25.2	
**48 hpi. *Cpn***	**Biological Replicate 1**	8.47	6.55
	**Biological Replicate 2**	2.02	
	**Biological Replicate 3**	3.84	
**72 hpi. UN**	**Biological Replicate 1**	0.804	15.78
	**Biological Replicate 2**	0.0684	
	**Biological Replicate 3**	6.32	
**72 hpi. *Cpn***	**Biological Replicate 1**	1.84	2.11
	**Biological Replicate 2**	0.497	
	**Biological Replicate 3**	0.322	

Table 2: %CV for soluble APPβ

		Intra-Assay CV %	Inter-Assay CV %
**6 hpi. UN**	**Biological Replicate 1**	3.59	0.04
	**Biological Replicate 2**	3.28	
	**Biological Replicate 3**	1.29	
**6 hpi. *Cpn***	**Biological Replicate 1**	2.21	0.09
	**Biological Replicate 2**	9.8	
	**Biological Replicate 3**	2.99	
**24 hpi. UN**	**Biological Replicate 1**	0.947	0.06
	**Biological Replicate 2**	2.81	
	**Biological Replicate 3**	0.342	
**24 hpi. *Cpn***	**Biological Replicate 1**	2.86	0.03
	**Biological Replicate 2**	5.9	
	**Biological Replicate 3**	3.49	

**48 hpi. UN**	**Biological Replicate 1**	2.81	0.04
	**Biological Replicate 2**	4.38	
	**Biological Replicate 3**	5.34	
**48 hpi. *Cpn***	**Biological Replicate 1**	1.48	0.02
	**Biological Replicate 2**	1.41	
	**Biological Replicate 3**	2.51	
**72 hpi. UN**	**Biological Replicate 1**	5.06	0.10
	**Biological Replicate 2**	10.5	
	**Biological Replicate 3**	1.13	
**72 hpi. *Cpn***	**Biological Replicate 1**	0.944	0.07
	**Biological Replicate 2**	6.83	
	**Biological Replicate 3**	2.68	
